# A Retrospective Cross-Sectional Analysis of Viral SARI in Pregnant Women in Southern Brazil

**DOI:** 10.3390/microorganisms12081555

**Published:** 2024-07-30

**Authors:** Sonia Maria Lissa, Bruna Amaral Lapinski, Maria Ester Graf, Somaia Reda, Maria do Carmo Debur, Mayra Presibella, Luciane Aparecida Pereira, Newton Sérgio de Carvalho, Jaqueline Carvalho de Oliveira, Sonia Mara Raboni, Meri Bordignon Nogueira

**Affiliations:** 1 Postgraduate Program in Tocogynecology and Women’s Health, Federal University of Parana Universidade Federal do Paraná, Curitiba 80060-900, Brazil; sonia.lissa@hc.ufpr.br (S.M.L.);; 2 Postgraduate Program in Internal Medicine and Health Science, Federal University of Parana, Curitiba 80060-900, Brazil; bruna.trilhas@gmail.com; 3 Epidemiology Division, Hospital do Trabalhador, Curitiba 81050-000, Brazil; mesthergraf@gmail.com; 4 Gynecology and Obstetrics Division, Hospital do Trabalhador, Curitiba 81050-000, Brazil; somaia.reda@gmail.com; 5 Public Health Laboratory, São José dos Pinhais 83060-500, Brazil; mariadebur@sesa.pr.gov.br (M.d.C.D.); mayrapresibella@hotmail.com (M.P.); 6 Virology Laboratory, Hospital de Clínicas, Federal University of Parana, Curitiba 80060-900, Brazil; lucianeapereira@yahoo.com.br; 7 Department of Tocogynecology, Federal University of Parana, Curitiba 80060-900, Brazil; 8 Department of Genetics, Federal University of Parana, Curitiba 80060-900, Brazil; jaqueline.carvalho@ufpr.br; 9 Infectious Diseases Division, Hospital de Clínicas, Federal University of Parana, Curitiba 80060-900, Brazil; sraboni@ufpr.br

**Keywords:** pregnant women, SARI, influenza, viral infections, respiratory infections

## Abstract

Pregnant women (PW) are at a higher risk of diseases and hospitalization from viral respiratory infections, particularly influenza and SARS-CoV-2, due to cardiopulmonary and immunological changes. This study assessed the impact of viral respiratory infections on PW hospitalized with severe acute respiratory infection (SARI) prior to the COVID-19 pandemic. It is a cross-sectional study with 42 PW and 85 non-pregnant women (NPW) admitted with SARI to two tertiary hospitals between January 2015 and December 2019. The rates of virus prevalence, SARI hospitalization, length of hospital stay, oxygen supplementation, intensive care unit (ICU) admission, and death were comparable between PW and NPW. A multivariate analysis showed that PW had a higher rate of viral SARI hospitalizations (OR = 2.37; 95% CI = 1.02–5.48) as compared to NPW, with the influenza virus being the most prevalent (aOR = 7.58; 95% CI = 1.53–37.66). The length of hospital stays (aOR = 0.83; 95% CI = 0.73–0.95) and admissions to the ICU (aOR = 0.028; 95% CI = 0.004–0.25) were lower in PW as compared to hospitalized NPW. The influenza virus had a greater impact on the frequency of SARI in the group of PW, and these had a better outcome than NPW due to the earlier antiviral treatment they received.

## 1. Introduction

Community-acquired pneumonia (CAP) is the leading cause of hospitalization and mortality worldwide **[1]**, with community respiratory viruses (CRVs) being responsible for 15–30% of cases [2]. Many of these cases are associated with severe acute respiratory illness (SARI), an acute respiratory infection that requires hospitalization and can be lethal [3].

Severe acute respiratory illness (SARI) comprises a set of symptoms, such as the following: O2 saturation <95% in room air, shortness of breath or increased respiratory rate assessed according to age, worsening of the clinical picture of the underlying disease, and hypotension in relation to the usual blood pressure of the patient or individual of any age with acute respiratory failure [4]. It is characterized by acute bilateral inflammatory pulmonary infiltrates and severe hypoxia [5]. It may have an infectious or non-infectious cause, including pneumonia, non-pulmonary sepsis, aspiration of gastric and/or oral esophageal contents, and severe trauma. It is a common cause of respiratory failure in critically ill patients and is defined by the acute appearance of non-cardiogenic pulmonary edema, hypoxemia, and the need for mechanical ventilation [6].

Among infectious causes, CRVs identified in SARI include influenza A and B (IF), human adenovirus (hAdV), respiratory syncytial virus (RSV), human rhinovirus (hRV), human metapneumovirus (hMPV), the parainfluenza virus group (PIV 1,2, 3,4), human enterovirus (hEV), human bocavirus (hBoV), and human coronavirus-hCoV (229E, OC43, HKU1, NL-63) [7]. Additionally, SARS-CoV-2 has emerged as a recent threat to public health [8]. CRV infections in pregnant women can result in a spectrum of mild to severe cases of acute respiratory illness [9].

Pregnancy is a serious risk factor for influenza and COVID-19, as physiological changes that occur during pregnancy—including decreased lung capacity, cell-mediated immunity, and increased oxygen consumption—predispose those women to worse outcomes than the general population [10]. Vaccination is an essential element of pre-pregnancy, prenatal, and postpartum care, as it should protect against the risk of complications and progression to SARI during the prenatal or postpartum period [11,12].

In the Brazilian A (H1N1) vaccination campaign in 2010, planned as a national program, the vaccine was mandatory in all public health institutions and especially in those that offered prenatal care [13]. Since 2016, the Brazilian Ministry of Health has extended the recommendation to children <5 years of age, pregnant women, people with chronic non-communicable diseases, and health professionals [14,15].

With the 2020 COVID-19 pandemic, immunization against SARS-CoV-2 has been a priority, and current recommendations from the World Health Organization, the Centers for Disease Control and Prevention, and professional organizations are for pregnant, postpartum, and breastfeeding women receive vaccination against SARS-CoV-2, as these women were designated as a risk group for SARI [11]. In 2021 in Brazil, the estimated case fatality rate of COVID-19 among pregnant and postpartum women was 7.2%, compared to 2.8% in the general population. Furthermore, SARS-CoV-2 has been associated with adverse perinatal events, such as premature birth, fetal loss, and neonatal mortality [16].

Despite the existence of immunization, treatment, and a global surveillance network for influenza viruses and SARS-CoV-2, studies on viral SARI in pregnant women remain limited [17,18]. In addition to these, only research on RSV in pregnant women has gained prominence recently; however, the focus is on the protection of newborns [19].

This study aimed to examine the burden of viral respiratory infections in pregnant women hospitalized with SARI prior to the COVID-19 pandemic.

## 2. Materials and Methods

A retrospective, cross-sectional study was carried out between January 2015 and December 2019 in two tertiary hospitals in Curitiba, Southern Brazil, using secondary data from medical record reviews.

All information on hospitalizations and deaths related to SARI is recorded in the Epidemiological Surveillance Information Service (SIVEP-Gripe) and forms mandatory notifications. SIVEP-Gripe records several variables, some of which include date of notification, onset of symptoms, hospitalization, clinical sample collection, detection, laboratory results, and case resolution. Access to the identified and non-duplicated data is publicly available (https://opendatasus.saude.gov.br/dataset/bd-srag-2019 accessed on 28 June 2024).

Both hospitals approved the study via their respective Ethics Committees under the identification numbers 15599.8.0000.0096 and 155.99119.8.3001.5225, respectively.

Sampling: The study was carried out with nasopharyngeal swabs collected 3 to 5 days from the onset of symptoms in patients admitted to both hospitals. The genetic material was extracted with the following commercial kits: Invitrogen PureLink, (Invitrogen, Carlsbad, CA, USA), High Pure Viral Nucleic Acid (Roche, Inc., Basel, Switzerland), and Viral RNA, Viral DNA Extraction (Promega, Madison, WI, USA), QIAsymphony automation equipment (Qiagen, Hilden, Germany), and MagNAPure96 equipment (Roche, Mannheim, Germany). Afterwards, the reverse transcription technique was performed, followed by the quantitative polymerase chain reaction (RT-qPCR) following the WHO protocol (2009) [20] as updated in 2017 [21].

Of the 1132 SARI cases reported to SIVEP (Epidemiological Surveillance Information Service) between 2015 and 2019 in both tertiary hospitals, 49 pregnant women (PW) and 159 non-pregnant women (NPW) were eligible. Of these, 42 (85.7%) PW and 85 (53.4%) NPW were included in the study; adhering to the inclusion and exclusion criteria, 42/49 PW and 85/159 NPW with SARS were requested for research on respiratory viruses.

Inclusion criteria*:* PW aged ≥ 18 years, at any gestational period, hospitalized with SARI, and investigated for the following community respiratory viruses (CRVs): influenza A and B, hAdV, RSV, hRV, hMPV, hPIV group, hEV, hBoV, hCoV HKU1, 229E, OC43, and NL63 via RT-qPCR. NPW aged between 18 and 45 years, without underlying diseases, and hospitalized with SARI with the same CRVs described above.

**Figure 1:**
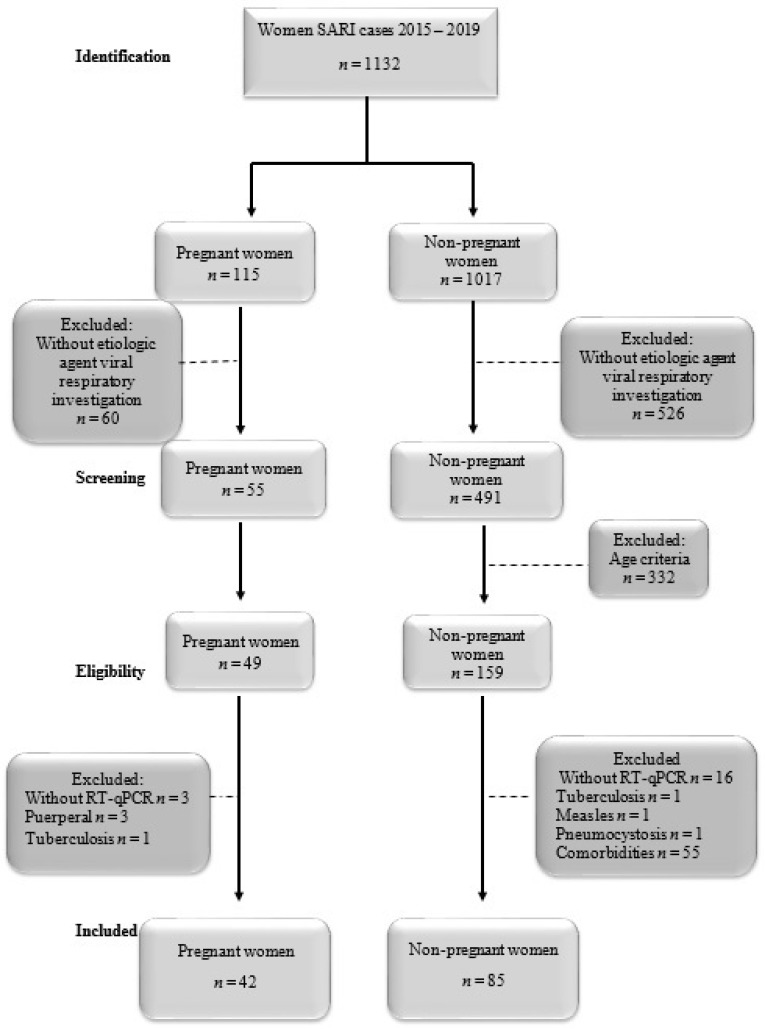
Flowchart of the study.

Exclusion criteria of PW with SARI: Seven were excluded: three due to lack of RT-qPCR, three for being postpartum, and one with a diagnosis of tuberculosis (Figure 1).

NPW with SARI: In total, 74 were excluded (46.5%): 16 due to lack of RT-qPCR, 1 with measles, 1 with pneumonia, 1 with a diagnosis of tuberculosis, and 55 with comorbidities (Figure 1). 

Definitions: Primary outcome: ICU admission. Secondary outcomes: length of hospital stay, ventilatory support, and death. Potential confounders: viral co-infection. Effect modifiers: influenza immunization and treatment, underlying diseases, and age group. Bias: etiologic agent unknown in negative RT-qPCRs.

Data analysis: Statistical analyses were performed using IBM SPSS version 23. Univariate analysis used Fisher’s exact and Chi-square tests for categorical variables and the Mann–Whitney and Kruskal–Wallis tests for continuous variables, as appropriate. Multivariate logistic regression analyses evaluated covariates related to outcomes. The adjusted odds ratio (aOR) was calculated using the multivariate model with a stepwise selection of variables, with a cutoff point of *p* < 0.2, and a Pearson’s analysis to test the model fit. Multivariate linear regression analysis was conducted to assess the impact of viral SARI on length of stay. All statistical tests were two-sided, with significance set at *p* < 0.05, and a confidence interval (CI) of 95%.

## 3. Results

Of the 1132 women with SARI cases between 2015 and 2019 in both tertiary hospitals, 60 PW and 526 NPW were excluded for not undergoing investigation for respiratory viruses. A total of 49 PW and 159 NPW were eligible to participate in this study. Of these, 42 (86%) PW and 85 (53%) NPW were included, as shown in Figure 1.

### 3.1. Clinical–Epidemiological Characteristics

**Figure 2:**
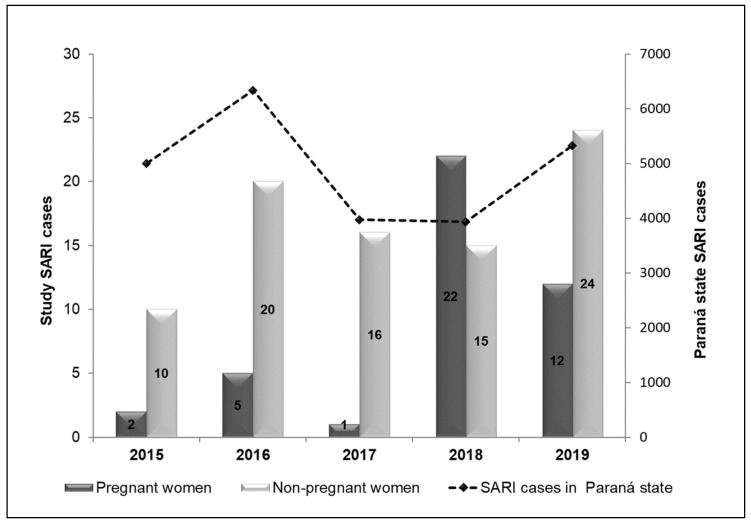
SARI cases among pregnant and non-pregnant women included in the study (primary axis) and the total number of SARI cases in the state of Paraná (secondary axis); absolute cases of SARI between 2015 and 2019. Legend: SARI, severe acute respiratory syndrome.

The SARI cases within the study period among PW were concentrated in 2018, while in NPW, they were distributed without much discrepancy, with 2015 having the lowest and 2019 having the highest number of cases, as depicted in Figure 2.

**Figure 3:**
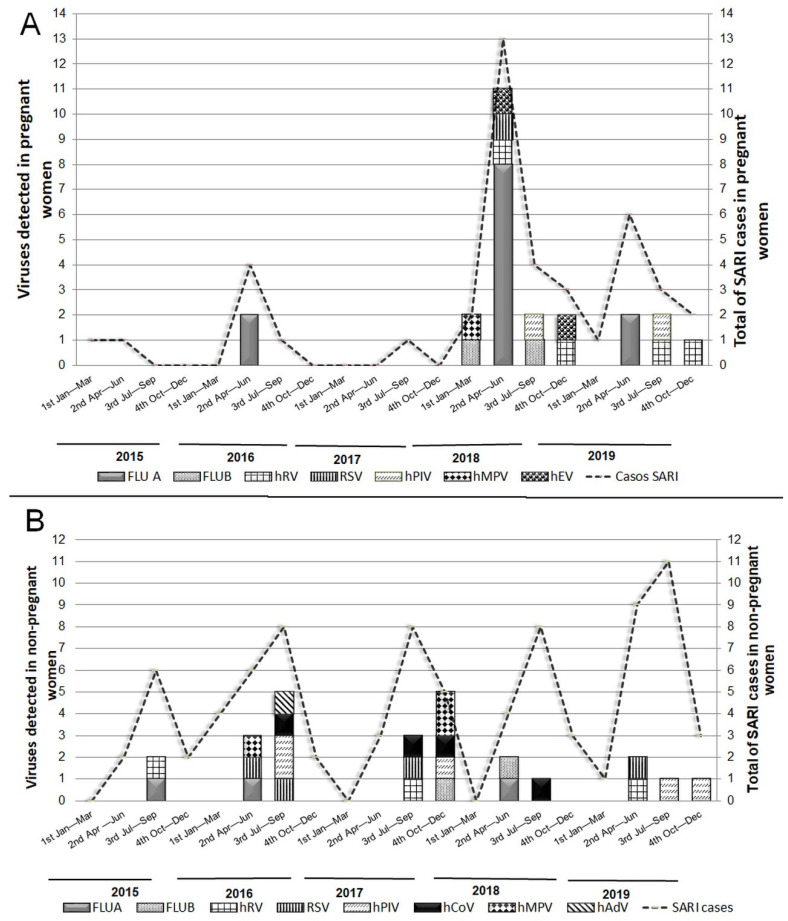
Viruses identified in pregnant (**A**) and non-pregnant women (**B**) between 2015 and 2019. Horizontal axis data were presented in the 4 annual quarters; 1st: January–March; 2nd: April–Jun; 3rd: Jul–September; 4th: October–December. Primary axis—number of viruses identified. Secondary axis—total of SARI cases in the group. FLU A: influenza type A; FLU B: influenza type B; hMPV: human metapneumovirus; hADV: human adenovirus; hPIV: parainfluenza virus; hRV: rhinovirus; RSV: respiratory syncytial virus; hCOV: coronavirus; hEV: enterovirus.

The peak of SARI cases in PW occurred during the coldest months (April–September), including viral SARI, which was concentrated in the second trimester of 2018 (April–Jun) (Figure 3A). The NPW showed similar seasonality, with more evenly distributed cases of SARI and viral SARI as compared to PW. However, the highest number of viral infections in NPW occurred in the third quarter (July–September) of 2016 and the fourth quarter (October–December) of 2017 (Figure 3B).

There was no difference in the number of PW hospitalized with SARI based on the gestational period. Only 45% of PW were immunized against influenza. Two-thirds of PW with asthma were infected with hRV, and one HIV-positive patient was infected with influenza. Of the five PW with comorbidities, only the HIV patients had been vaccinated against influenza. Antiviral treatment was prescribed more frequently in PW than in NPW (*p* < 0.0001). All PW with viral SARI (21/21) and 10/24 of NPW received antiviral therapy. A multivariate analysis showed that hospitalization rates for viral SARI were higher for PW than for NPW (aOR = 2.37; 95% CI = 1.02–5.48; *p* = 0.044), as shown in Table 1.

### 3.2. Impact of Viral SARI on PW and NPW

The median age of PW with viral SARI was similar to that of NPW. Of the 127 (42 PW + 85 NPW) SARI cases, 45 (35%) were positive for viruses. Of these, 50% (21/42) were PW and 28% (24/85) were NPW. Among the 14 viruses investigated via RT-qPCR, hBoV, hCoV, and hAdV were not identified in the PW group, and hEV was recognized only in the PW group.

Out of the 21 PW with viral SARI, 14 (67%) were infected with influenza and 10 (71.4%) did not receive the influenza vaccine. Of the four that received the vaccine, two received the vaccine less than 30 days before the infection, two were infected with influenza B, which was absent in the trivalent vaccine, and only one of the PW diagnosed with influenza SARI had been adequately immunized.

There was no difference in the median time of symptom onset and hospitalization between groups; the medical records of 95% (*n* = 20/21) of the PW and only 71% (*n* = 17/24) of the NPW were available. Three PW were co-infected: two with hRV, and only one with SARI had influenza and an RSV co-infection, was admitted to the ICU, had no comorbidities, and was not vaccinated. No cases of hCoV infections (HKU, OC43, and NL63) were detected.

In the NPW group, 24 were positive for viral SARI. The distributions of viral etiological agents were more homogeneous. Influenza was responsible for five (20.8%), and the other CRVs were detected in 15 patients (Table 2). Among these, one PIV + RSV co-infection was identified, and only hEV was not identified in the NPW group.

The logistic regression showed that the likelihood of viral SARI via influenza was seven times greater in PW than in NPW (aOR = 7.58; 95% IC = 1.53–37.66; *p* = 0.013).

However, the risk of NPW being admitted to the ICU due to viral SARI was 16 times higher than that of PW (aOR = 0.028; 95% IC = 0.004–0.25; *p* = 0.001). Nonetheless, there was no difference in median age or co-infection rates in the PW group. Linear regression showed a 16-day increase in the length of hospital stay for viral SARI in the NPW group (β = 16.38; 95% IC = 0.57–32.18; *p* = 0.043); see Table 2.

## 4. Discussion

In this study, influenza-associated viral SARI had a more significant impact on the number of hospitalizations among PW than NPW, similar to that found by Creanga et al., 2010, where pregnant women had a higher hospitalization rate and were more likely to have serious illnesses after the first trimester [22].

In Brazil, after the 2009 influenza A(H1N1) pandemic, pregnant women considered part of a risk group received greater attention, as they were susceptible to developing SARI with high morbidity and increased risk of death [13,23,24].

A series of studies in Brazil have highlighted the significant impact of SARI, particularly in the context of the COVID-19 pandemic. Bastos et al. (2020) [25] and Niquini et al. (2020) [26] found an increase in hospitalizations for SARS due to COVID-19 in people with comorbidities, indicating a potential for a more severe progression of the disease. Leal et al. (2021) [27] highlighted the high mortality among pregnant women with SARS due to COVID-19. These findings highlight the importance of continued surveillance and specific interventions to mitigate the impact of viral SARI, especially in vulnerable populations.

Influenza vaccine coverage, the rate of neuraminidase inhibitor prescriptions, and early hospitalization likely explain these findings [18,19].

The distribution of total SARI cases followed influenza seasonality in both groups [28]. The viral SARI rate among PW was higher than NPW, consistent with the findings from the state of Paraná. Influenza was the most frequent among the investigated viruses in PW, followed by hRV, with a similar profile as described previously [9,29]. In accordance with Azziz-Baumgartner et al. (2021) [30], we did not identify any cases of hAdV among PW.

The influenza vaccine coverage in PW was below the target recommended by immunization policies, ranging from 58 to 76% [19] (MS, 2021). The low vaccination coverage observed was consistent with current studies that reported vaccination rates between 5 and 58% among PW [30,31], with an average of 59% for the American continent according to the Pan American Health Organization (PAHO) [32].

Vaccination is the main public health measure used to reduce the frequency of severe influenza cases [14]. As of 2009, the benefits of influenza vaccination were observed not only for pregnant women but also for newborns during the first six months of life [15]. Vaccination with either the seasonal or pandemic vaccine has been shown to be cost-effective in pregnancy. After vaccination, pregnant women had protective concentrations of anti-influenza antibodies, conferring immunogenicity to newborns [33].

The prevalence of comorbidities among PW was lower than previously described (approximately 30%), with asthma being the most frequent, followed by diabetes and hypertension [34]. Although antiviral treatment should be prescribed for all SARI patients [35], prescription was higher for PW than for NPW, especially among those with viral SARI, for which all PW were treated, unlike the other group, which was not attended to in time to institute treatment because it was not a priority [36].

PW were twice as likely to be hospitalized for viral SARI than NPW, with influenza being the main cause of viral SARI, as described previously [37] and confirmed in the present study. However, meta-analysis studies have shown that despite the increase in the need for hospitalization, pregnancy was not associated with more serious flu-associated outcomes, such as ICU admission and death [38,39]. Consistent with our findings, Mertz et al. (2019) found a seven-fold increased risk of influenza SARI among PW compared to NPW. PW with viral SARI also had a decreased length of hospital stay, a finding not previously described.

During the (H1N1)pdm09 pandemic, no differences were found in histopathology between severe cases of PW and NPW women [40]. Although Littauer et al. (2017) demonstrated in a mice model that pregnancy reduces viral clearance in the lungs, they did not observe any difference between the expression of inflammatory cytokines and chemokines in the lungs of PW and NPW mice [41,42].

Prophylaxis, treatment, and clinical management measures may also explain our findings. We observed that 71.4% of PW with influenza did not receive the flu vaccine in the present study. A recent meta-analysis study reported that immunization prevented 50–70% of influenza infections and 45–65% of worse outcomes [43]. However, adherence to vaccination by PW faces obstacles due to a lack of knowledge regarding the risks of influenza or the benefits of vaccination [44]. According to the Centers for Disease Control and Prevention (CDC) (2017), 21% of PW did not receive influenza vaccine recommendations from doctors or medical staff [45].

Antiviral treatment has also been shown to reduce the risk of worse outcomes, including the length of hospital stay, ICU admission, and death in PW [46,47]. In this study, all PW with viral SARI received antiviral treatment. However, our data on the prescription of antivirals suggest that the clinical management of PW is more precise, targeting interventions in the early stages of the infection, unlike what is observed with NPW, who are clinically managed late, with a risk of worse results [18]. Although there are national guidelines for the clinical management of SARI [35], some hospital protocols may differ depending on their participation in the influenza surveillance network or the availability of hospital beds. As NPW are not a risk group for the development of SARI [18], the majority of these patients tend to be hospitalized in more advanced stages of the disease in a tertiary hospital; this is in addition to the lack of clinical–epidemiological data available due to the retrospective collection of data in medical records. All these data reinforce the findings from the present study, emphasizing the importance of investigating factors associated with the clinical evolution and severity of viral SARI in pregnant women, in order to promote public health decisions in health programs and specific clinical management protocols for this group.

This study had some limitations: the small number of participants due to the specificity of the selected group and the large number of SARI notification forms with incomplete data, including substantial under-reporting. Even so, the data presented in this study are extremely relevant and could serve as a basis for expanded studies and for decision-making in public policies to reinforce the recommendation of immunization for pregnant women to prevent SARI due to influenza.

## 5. Conclusions

The impact of the influenza virus on the number of SARI hospitalizations is usually greater in pregnant women (PW) compared to non-pregnant women (NPW). However, this study highlights an inverse association with disease severity, emphasizing the importance of early antiviral treatment. Additionally, this work underscores the need for new strategies to increase vaccination coverage and ensure accurate clinical management and treatment to reduce the SARI viral burden in pregnant women.

## Figures and Tables

**Table 1:** Clinical–epidemiological characteristics of pregnant and non-pregnant women groups hospitalized for SARI from 2015 to 2019.

	Pregnant Women *n* = 42	Non-Pregnant Women *n* = 85	*p*	aOR (95% IC)	*p*
Gestational quarter n (%)					
1st trimester	13 (31)	NA	-		
2nd trimester	16 (38)	NA			
3rd trimester	13 (31)	NA			
Age median—years (IQR)	28 (22–32)	31 (24–36)	0.011		
Age group—years, n (%)					
18–27	21 (50)	26 (31)	0.035		
28–37	18 (43)	40 (47)			
38–45	3 (7)	19 (22)			
Ethnic group n (%)					
Caucasian	34 (81)	77 (90)	0.091		
African descendant	8 (19)	8 (10)			
Influenza vaccine n (%)					
Yes	19 (45)	0	-		
No	18 (43)	85 (100)			
Not described	5 (12)	0			
Underlying diseases n (%)	5 (12)	0	-		
Antiviral treatment n (%)	37 (88)	35 (41)	<0.0001		
Symptoms					
Fever	31 (74)	58 (68)	0.545		
Cough	37 (88)	56 (66)	0.010		
Sore throat	14 (33)	9 (11)	0.459		
Dyspnea	30 (71)	57 (67)	0.553		
Viral *SARI n (%)	21 (50)	24 (28)	0.019	2.37 (1.02—5.48)	0.044

OR: Odds Ratio; IC: confidence interval*;* IQR: interquartile range; * SARI: severe acute respiratory syndrome.

**Table 2:** Impact of viral SARI on pregnant and non-pregnant women groups hospitalized from 2015 to 2019.

	Pregnant Women *n* = (21/42)	Non-Pregnant Women *n* = (24/85)	*p*	aOR (95% IC)	*p*
Median age—years (IQR)	27 (24–33)	31 (25–36)	0.270		
Median time symptoms elapsed until hospitalization—days (IQR)	2 (1.0–3.0)	2 (1.0–6.5)	0.417		
Viral coinfection n (%)	3 (14)	1 (4)	0.318		
Viruses detected n (%)					
FLU (A and B)	14 (67)	5 (21)	0.002	7.58 (1.53–37.66)	0.013
hMPV	1 (5)	3 (12)	0.614		
hAdV	0	1 (4)	-		
hPIV (1,2,3, and 4)	2 (10)	5 (21)	0.428		
hRV (A, B, and C)	4 (19)	3 (12)	0.686		
RSV (A and B)	1 (5)	4 (17)	0.357		
hCoV (HKU1/229E/OC43/NL63)	0	4 (17)	0.239		
hEV	2 (10)	0	-		
Primary outcomes					
Length of stay—days (IQR)	3 (2–5)	14 (5–20)	0.0004	0.83 (0.73–0.95)	0.006
Ventilatory support n (%)	8 (38)	14 (58)	0.236		
ICU admission n (%)	1 (5)	16 (67)	<0.0001	0.03 (0.00–0.25)	0.001
Death n (%)	0	1 (4)	-		

Legend: OR: Odds Ratio; IC: confidence interval; IQR: interquartile range: ICU: intensive care unit; FLU A: influenza type A; FLU B: influenza type B; hMPV: human metapneumovirus; hADV: human adenovirus; hPIV: parainfluenza virus; hRV: rhinovirus; RSV: respiratory syncytial virus; hCOV: coronavirus; hEV: enterovirus.

## Data Availability

The data are contained within the article.
